# Description of the Soil Diatom *Sellaphora terrestris* sp. nov. (Bacillariophyceae, Sellaphoraceae) from Vietnam, with Remarks on the Phylogeny and Taxonomy of *Sellaphora* and Systematic Position of *Microcostatus*

**DOI:** 10.3390/plants11162148

**Published:** 2022-08-18

**Authors:** Anton Glushchenko, Elena Kezlya, Yevhen Maltsev, Sergei Genkal, John Patrick Kociolek, Maxim Kulikovskiy

**Affiliations:** 1 K.A. Timiryazev Institute of Plant Physiology RAS, IPP RAS, 35 Botanicheskaya St., 127276 Moscow, Russia; 2 Papanin Institute for Biology of Inland Waters RAS, IBIW RAS, Borok, 152742 Yaroslavl Region, Russia; 3 Museum of Natural History, Henderson Building, 15th and Broadway, Boulder, CO 80309, USA

**Keywords:** diatoms, morphology, molecular investigation, 18S rDNA, *rbc*L, soil, southeast asia, Cát Tiên National Park

## Abstract

In material isolated from soils of Cát Tiên National Park, we isolated four strains that were assigned to the genus *Sellaphora*. Identification was carried out on the basis of morphological and molecular studies. We proposed a new species named *Sellaphora terrestris* sp. nov. An evolutionary distance matrix, based on the 18S rDNA gene including the V4 domain, showed the new species shared 94.1–97.2% similarities with other *Sellaphora* sequences. The new species is morphologically similar to species previously identified as representatives of the genus *Microcostatus*.

## 1. Introduction

The genus *Sellaphora* Mereschkowsky was described in 1902 [1]. It has over 250 species [2,3]. It is the largest genus in the family Sellaphoraceae, which also includes the genera *Buryatia* Kulikovskiy, Lange-Bertalot, and Metzeltin; *Caponea* Podzorski; *Dimidiata* Hajos; *Diprora* Main; *Eolimna* Lange-Bertalot and Schiller; *Fallacia* Stickle and D.G. Mann; *Lacuneolimna* Tudesque, Le Cohu, and Lange-Bertalot; *Okhapkinia* Glushchenko, Kulikovskiy, and Kociolek; and *Rossia* Voigt [2,3,4,5]. Currently, species of the genus *Sellaphora*, relative to other genera of the family, are the best studied using morphological, cytological, reproductive, and molecular methods [6,7,8,9,10,11,12].

The monophyly of *Sellaphora* has been well supported in a number of studies [6,9,13,14,15]. Currently, there are 550 sequences for 39 taxonomic units of *Sellaphora* deposited in GenBank (data on 1 August 2022), of which 17 have been identified to the species level, four taxa have been identified presumptively, and 18 have not been identified.

*Sellaphora pupula* is the type species of the genus [16]. The species complex of *S. pupula* (Kützing) Mereschkowsky (230 sequences in GenBank; data on 1 August 2022) has been the most studied taxa within the genus. It is a common and frequently reported species and has long been classified as a taxon of high morphological variability [17,18], resulting in many variations being described [19]. Study of the molecular structure of this species complex has been primarily related to questions of species boundaries, the biological significance of variations, the study of cryptic and pseudocryptic diversity, biogeography, relationships of phylogeny and morphometric characters, and problems of speciation [6,8,12,20,21]. This species complex was also used as a convenient model for testing the resolution of various genetic markers [9,22]. As a result, it has been shown that “*S. pupula* (*Navicula pupula* sensu Hustedt [17,23]; Krammer and Lange-Bertalot [24]) represents a paraphyletic group” [12], while morphological synapomorphies for species separation have not been clearly defined. The results of experimental mating tests indicate that clones of different populations from the same reservoir with differences in size, valve shape, or striae density (so-called demes) are not compatible [6,13,16,25]. On the contrary, when studying the molecular structure of the genes of *S. auldreekie* D.G. Mann and S.M. McDonald, *S. bisexualis* D.G. Mann and K.M. Evans, and *S. capitata* D.G. Mann and S.M. McDonald, it was shown that the sequences of strains obtained from very distant places with different climates (tropical in Australia and temperate in UK) were identical [15,20,26], which indicates a wide distribution of individual genetic lines.

The genus is distributed all over the world, inhabiting different types of water bodies and soils [4,27,28]. All *Sellaphora* clones studied to date have been isolated from fresh water bodies, and the vast majority of strains were obtained in Europe: UK (Scotland), Belgium, Germany, and Czech Republic [9,12,20,21,22]. No more than four strains of *Sellaphora* have been deposited in GenBank from Japan, South Korea, Mexico, and New Zealand (subtropics); Tanzania and East Africa (subequatorial climate); and USA, Dakota country, located in Minnesota (continental climate). From tropical regions, 19 *Sellaphora* strains have been studied in water bodies of Australia [9,20,22] and one in the Hawaiian Islands [29]. Soil strains for representatives of this genus using molecular approaches have not yet been studied. At the same time, as noted in the work of [30], devoted to the study of *Coccomyxa* Schmidle, the ecological characteristics of the species (such as living stage and habitat) are in good agreement with the phylogeny (and may be the basis for the identification of the species) if the lineage is well maintained. At the same time, the acquisition of molecular sequences, along with well-studied morphology, plays an important role in the accumulation of data for the correct identification of taxa using high-throughput sequencing (HTS) methods.

The aerophilic diatom flora of Southeast Asia are not well studied. Our work has focused on the diatoms of soils of Cát Tiên National Park. Work has been carried out on the composition and structure of forest soil algocenoses in this national park [31]. In addition, species from the genera *Mayamaea* Lange-Bertalot and *Placoneis* Mereschkowsky were described from the soils of this national park [32,33]. However, a targeted study of *Sellaphora* species from the soils of Southeast Asia had not been attempted previously. For comparison, we can only point to the species described by Friedrich Hustedt from Indonesia, which currently belong to the genus *Sellaphora*: *S. subbacillum* (Hustedt) Falasco et al. [34] and *S. perventralis* (Hustedt) Tuji [35]. However, *S. subbacillum* (as *Navicula subbacillum* Hustedt) was noted as an aerophilic species [36]. Thus, we can conclude that the soil diatom flora of Southeast Asia requires systematic investigation.

## 2. Results

### 2.1. Taxonomic Analysis

**Figure 1:**
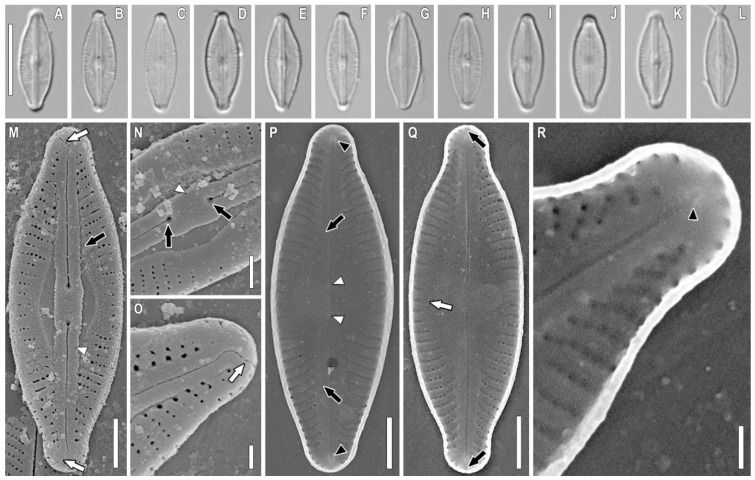
*Sellaphora terrestris* sp. nov. Strain VP 304. Slide no. 07062a. (**A**–**L**). Light microscopy, differential interference contrast, size diminution series (**M**). Scanning electron microscopy, external views. (**M**–**O**). Scanning electron microscopy, internal views. (**P**–**R**). Black arrow shows the longitudinal grooves in the axial area. White arrows show the distal raphe ends. White arrowhead shows the conopeum. (**N**). Black arrow shows the proximal raphe ends. White arrowhead shows the conopeum. (**O**). White arrows show the distal raphe end. (**P**). Black arrow shows the axial area. Black arrowheads show the helictoglossae. White arrowheads show the central raphe ends. (**Q**). Black arrows show the rounded apical pits. White arrow shows the areolae covered by hymenes (**R**). Black arrowhead shows the helictoglossa. (**G**). Holotype. Scale bar (**A–L**) = 10 μm; (**M,P,Q**) = 2 μm; (**N**) = 1 μm; (**O,R**) = 0.5 μm.

**Figure 2:**
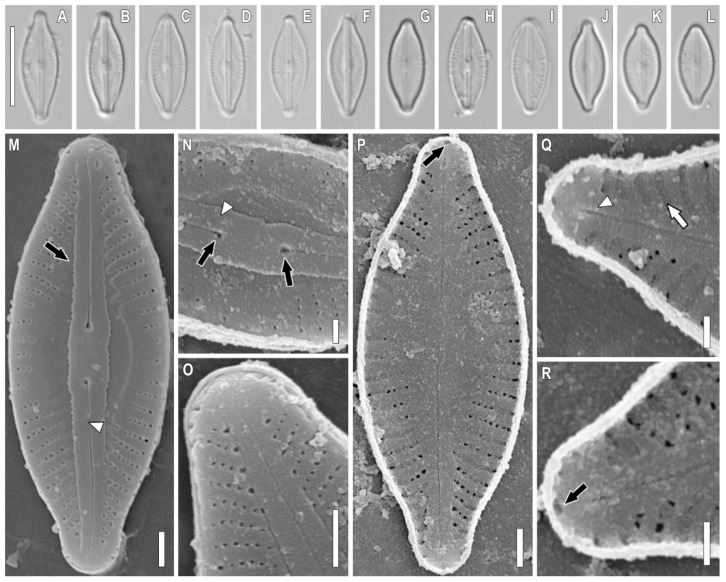
*Sellaphora terrestris* sp. nov. Strain VP 272. Slide no. 07030. (**A**–**L**). Light microscopy, differential interference contrast, size diminution series (**M**–**O**). Scanning electron microscopy, external views. (**P**–**R**). Scanning electron microscopy, internal views. (**M**). Black arrow shows the longitudinal grooves in the axial area. White arrowhead shows the raphe. (**N**). Black arrow shows the proximal raphe ends. White arrowhead shows the conopeum. (**P**). Black arrow shows the rounded apical pit. (**Q**). White arrowhead shows the helictoglossa. (**R**). Black arrow shows the rounded apical pit. Scale bar (**A**–**L**) = 10 μm; (**Q**) = 2 μm; (**M**,**O**,**P**) = 1 μm; (**N**,**R**,**S**) = 0.5 μm.

**Figure 3:**
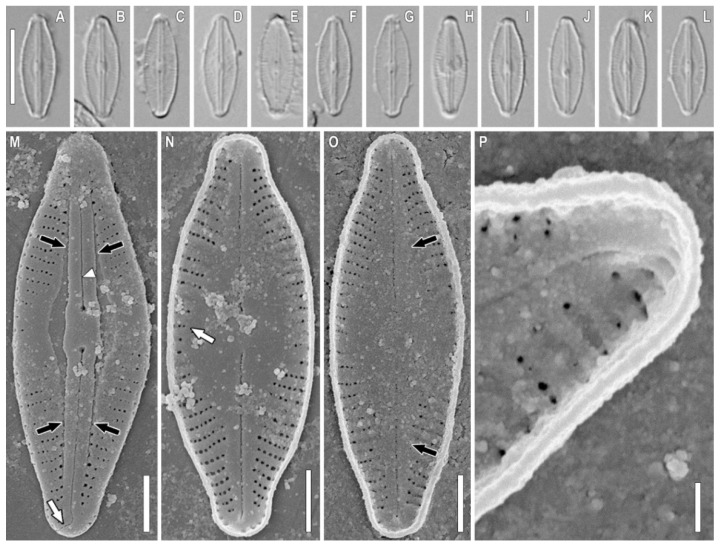
*Sellaphora terrestris* sp. nov. Strain VP 303. Slide no. 07061. (**A**–**L**). Light microscopy, differential interference contrast, size diminution series (**M**). Scanning electron microscopy, external views. (**N**–**P**). Scanning electron microscopy, internal views. (**M**). Black arrow shows the longitudinal grooves in the axial area. White arrows show the distal raphe end. (**N**). White arrows show the striae. (**O**). Black arrow shows the axial area. Scale bar (**A**–**L**) = 10 μm; (**M**–**O**) = 2 μm; (**P**) = 1 μm.

**Figure 4:**
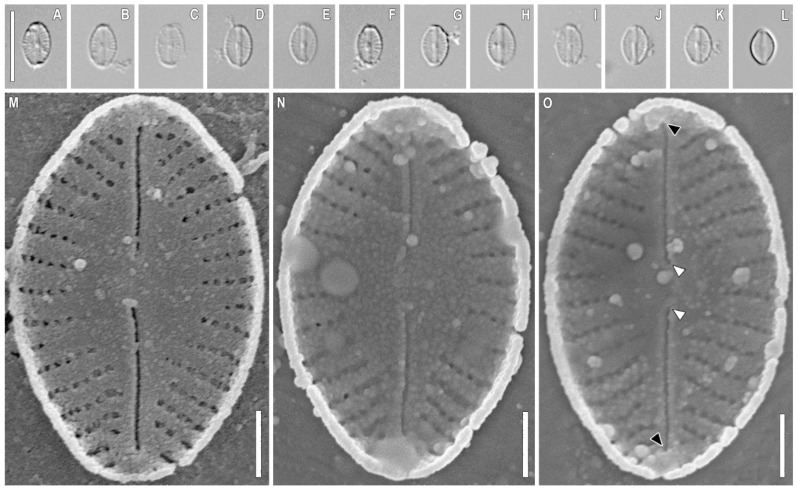
*Sellaphora terrestris* sp. nov. Strain VP 299. Slide no. 07057. (**A**–**L**). Light microscopy, differential interference contrast, size diminution series (**M**–**O**). Scanning electron microscopy, internal views. Black arrowheads show the helictoglossae. White arrowheads show the central raphe ends. Scale bar (**A**–**L**) = 10 μm; (**Q**) = 2 μm; (**M**–**O**) = 1 μm.

*Sellaphora terrestris* Glushchenko, Kezlya, Maltsev, and Kulikovskiy sp. nov. (Figure 1, Figure 2, Figure 3 and Figure 4).

**Holotype here designated:** Slide no. 07062a (Figure 1G), from oxidized culture strain B703, isolated from the sample 195, deposited in herbarium of MHA, Main Botanical Garden Russian Academy of Science, Moscow, Russia.

**Isotype.** Slide no. 07062b, Collection of Maxim Kulikovskiy at the Herbarium of the Institute of Plant Physiology Russian Academy of Science, Moscow, Russia.

**Reference strain.** VP304, isolated in from the sample no. Kt59, deposited in the collection of Maxim Kulikovskiy at the Herbarium of the Institute of Plant Physiology Russian.

Academy of Science, Moscow, Russia.

Representative specimens. Strain VP304 (slide no. 07062a), VP272 (slide no. 07030), VP303 (slide no. 07061), VP299 (slide no. 07057); sample Kt33 (slide no. 07223), Kt59 (slide no. 06989), Kt60 (Slide no. 07232).

**Type locality.** Southeast Vietnam, Cát Tiên National Park, agricultural field soil surface, 11°24.241′ N, 107°22.468′ E.

**Sequence data**. Partial 18S rDNA gene sequences comprising V4 domain (GenBank accession numbers: ON332057 for VP304, ON332054 for VP272, ON332055 for VP299, ON332056 for VP303) and partial *rbc*L sequences (GenBank accession numbers: ON350766 for VP304, ON350763 for VP272, ON350764 for VP299, ON350765 for VP303).

**Etymology.** The specific epithet “*terrestris*” refers to the terrestrial habitat from where the new species was extracted.

**Distribution.** So far only known only from the Cát Tiên National Park (agricultural and forest soils).

**Comment.** We investigated the four strains of *S. terrestris* (VP304, VP272, VP299, VP303) and found that there were some morphological differences expressed across the strains. In particular, strain VP299 (Figure 4) has smaller valves, almost elliptical, without distinct apices (length 5.2–6.9 μm and width 3.7–4.0 μm) in comparison with VP304, VP303, and VP272 (length 11–14.3 μm and width 4.5–5.1 μm). Apparently, the strain VP299 presented the smallest valves during the valve diminution series. Despite these differences, all of these strains form one branch with maximum statistical support on the phylogenetic tree.

**Description.** LM (Figure 1A–L, Figure 2A–L, Figure 3A–L, and Figure 4A–L). The chloroplast structure was not observed. Valves linear-elliptical to elliptical with weakly convex margins. Ends are short rostrate to broadly rounded. Length 6.9–17.1 µm, width 3.7–6.1 µm. Axial area narrow, weakly extended to the central area. Central area small, longitudinally lanceolate. Raphe filiform, straight. Proximal raphe ends weakly expanded. Central raphe ends poorly visible in LM. Striae weakly radiate, almost parallel at the central part of valve, 28–35 in 10 µm. Areolae not resolved in LM.

SEM, external view (Figure 1M–O, Figure 2M–O and Figure 3M). Axial area forming two longitudinal grooves, which expand from the ends of the valves to their central part (Figure 1M, Figure 2M, and Figure 3M, black arrows). Raphe is straight, lying in raised, slightly asymmetrical conopeum (Figure 1M,N, Figure 2M,N and Figure 3M, white arrowheads). Proximal raphe ends almost straight, drop-shaped (Figure 1N and Figure 2N, black arrows). Distal raphe ends bent to one side and extending to the valve mantle (Figure 1M,O and Figure 3M, white arrows). Areolae rectangular to rounded, 60–65 in 10 µm.

**Figure 5:**
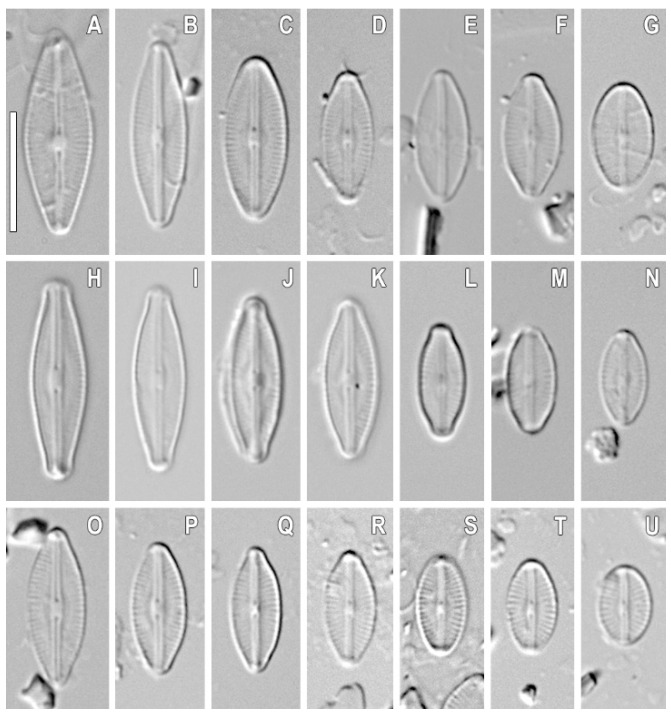
*Sellaphora terrestris* sp. nov. Light microscopy, differential interference contrast, size diminution series. Samples of wild populations. (**A**–**G**). Sample Kt33. Slide no 07223. (**H**–**N**). Sample Kt59. Slide no 06989. (**O**–**U**). Sample Kt60. Slide no 07232. Scale bar = 10 μm.

**Figure 6:**
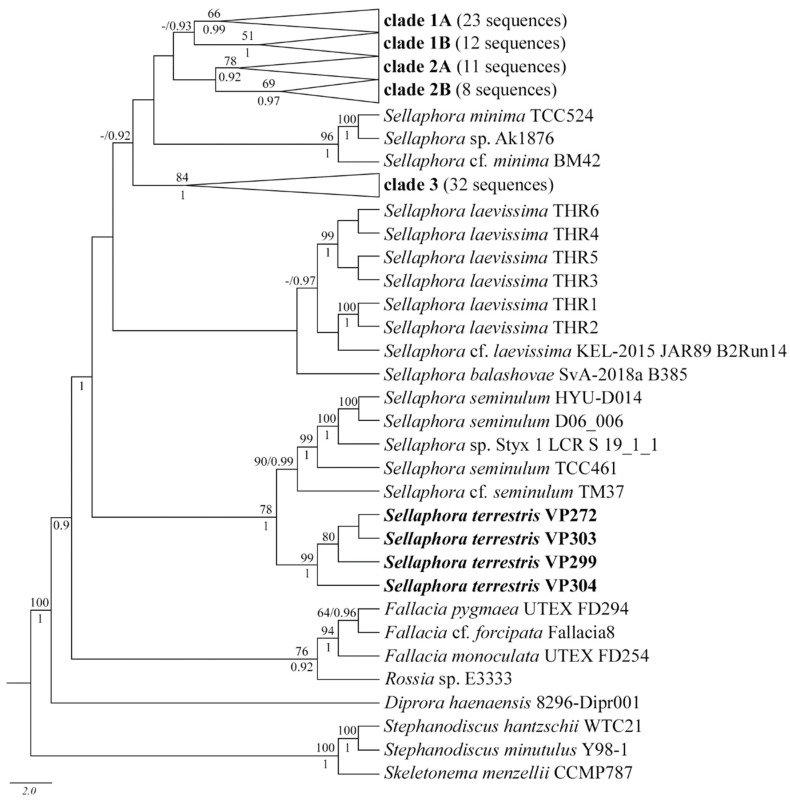
Phylogenetic position of *Sellaphora terrestris* sp. nov. (indicated in bold) within the Sellaphoraceae based on Bayesian inference for the 114 partial *rb*cL gene. The total length of the alignment is 957 characters. Values above the horizontal lines are bootstrap support the from ML analyses (<50 are not shown); values below the horizontal lines (or to the right of the slash) are Bayesian posterior probabilities (<0.9 are not shown). Strain numbers (if available) are indicated for all sequences. Clades indicated sensu Evans et al. [9], and Mann and Poulíčková [12] are in bold.

SEM, internal view (Figure 1P–R, Figure 2P–R, Figure 3N–P and Figure 4M–O). The axial area is quite wide, moving into an even wider central area (Figure 1P and Figure 5O, black arrows). The striae in the central part are noticeably shortened, up to 3–5 areolae per striae (Figure 1Q and Figure 3N, white arrows). Areolae covered by hymenes (Figure 2Q, white arrow). Raphe filiform, central raphe ends unilaterally slightly deflected (Figure 1P; 6O, white arrowheads), distal raphe ends curved in opposite directions and terminate as small helictoglossae (Figure 1P and Figure 6O, black arrowheads). Rounded apical pits are present at both apices (Figure 1Q and Figure 2P,R, black arrows).

**Figure 7:**
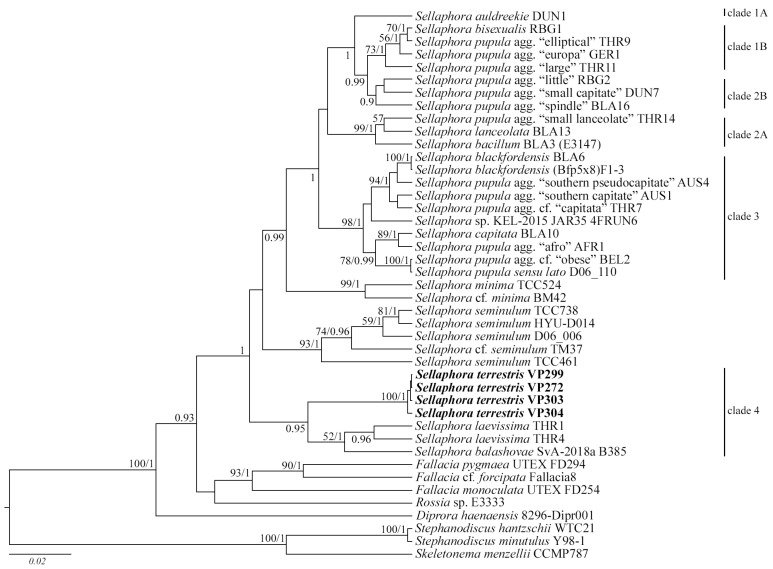
Phylogenetic position of *Sellaphora terrestris* sp. nov. (indicated in bold) within the Sellaphoraceae based on Bayesian inference for a concatenated alignment of 43 partial *rbc*L and partial 18S rDNA sequences. The total length of the alignment was 1361 characters. Values above the horizontal lines are bootstrap support from ML analyses (<50 are not shown); values below the horizontal lines (or to the right of the slash) are Bayesian posterior probabilities (<0.9 are not shown). Strain numbers (if available) are indicated for all sequences. Clades indicated sensu Evans et al. [9], and Mann and Poulíčková [12] are at the right.

Variability of the morphology of our species both in the natural material and in culture was noted (Table 1, Figure 5). This variability is expressed mainly in the shape of the valve ends. The cultivated strains are distinguished by having narrow valve apices. However, the ends of the valves range from beak-shaped (in large specimens) to broadly rounded (in small specimens). The shape of the valves themselves varies from linear-lanceolate and linear-elliptical to elliptical. At the same time, such features as the shape of the central and axial areas are the same. Quantitative characteristics in natural populations and images from cultures are quite similar. The length of valves in natural samples was 6.9–17.1 µm; in culture, it was 5.2–14.3 µm. The width of valves in natural samples was 4.2–6.1 µm; in culture, this parameter was 3.7–5.1 µm. Striae density in specimens from nature was 29–32 µm; in culture, the striae density was 28–35 µm (Table 1). Despite changes in the shape of the valves of the species introduced into culture, molecular genetic analysis allowed us to reliably establish the identity of the studied strains (Figure 6 and Figure 7). 

### 2.2. Molecular Analysis

Phylogenetic relationships within the genus *Sellaphora* were reconstructed using the maximum likelihood (ML, where LB is the bootstrap value) and Bayesian (Bayesian inference, where PP is the posterior probability) methods. The analysis used two approaches to determine the position of new *Sellaphora* soil strains: building trees based on the *rbc*L gene, with the inclusion of all relevant nucleotide sequences available in GenBank (a total of 106 *Sellaphora* strains); and tree construction based on pooled *rbc*L and 18S rDNA gene alignments (35 *Sellaphora* strains in total). In both cases, as in previous studies on the phylogeny of *Sellaphora* strains, most of the sequences included in the analysis formed several well-supported clades (clades 1–3, Figure 6).

The strains of *S. balashovae* Andreeva, Kulikovskiy and Kociolek; *S. laevissima* (Kützing) D.G. Mann; *S. minima* (Grunow) D.G. Mann; and *S. seminulum* (Grunow) D.G. Mann remained outside these clades. In the *rbc*L tree, the studied soil strains of *Sellaphora* formed a single subclade with high statistical support (99 LB, 1.0 PP). This subclade is located in the basal part of the *Sellaphora* tree and was not included in the previously described clades 1–3. At the same time, *S. seminulum* strains, with the support of 78 LB, 1.0 PP act as a sister subclade. The addition of the 18S rDNA gene to the sequence analysis slightly changed the topology of the phylogenetic reconstruction, while the composition of clades 1–3 remained the same. It should be noted that the position of the *S. minima* strains was different between the two analyses. If in the *rbc*L tree they were between clades 2 and 3, then in the *rbc*L and 18S rDNA tree they are outside clades 1–3 (Figure 7). Moreover, in the extended reconstruction, soil strains of *Sellaphora* moved away from strains of *S. seminulum* and formed a single clade with strains of *S. laevissima* (Figure 7).

Partial ribosomal 18S rDNA gene fragments comprising the V4 domain of the new soil *Sellaphora* strains showed 100% similarity. The VP304 strain was selected for further comparison of the percentage similarity. The evolutionary distance matrix based on the partial 18S rDNA gene showed that the VP304 strain shared 94.1–97.2% similarities with the other *Sellaphora* strains (Table 2), and 96.7% and 97.2% similarities with the closest strains in the phylogenetic tree (*S. laevissima* THR1, *S. laevissima* THR4, Figure 7). Within clade 1B, similarity was noted at the level of 99.5–100%. *Sellaphora* strains within clade 2A are characterized by 98.2–99.2% similarity, with clade 2B having 99.0–99.7%. The greatest differences between *Sellaphora* strains were noted within clade 3, with up to 2.7% (Table 2). In general, strains VP304, THR1, THR4, and B385 had a high similarity of the 18S rDNA gene region (97.2–99.5%), which allowed us to propose a new clade 4 within the genus *Sellaphora* based on these strains, and which has a separate position on the phylogenetic tree of the *rbc*L and 18S rDNA genes (Figure 2). The only strains of *Microcostatus* sp. (MMDL 54701 and MMDL 54702) differed significantly, by 6.9%, according to the 18S rDNA gene sequence. At the same time, the differences with the available sequences of the 18S rDNA gene of *Sellaphora* strains reached 14.6%, which may indicate either a distant relationship with *Sellaphora* strains, or incorrect identification of strains MMDL 54701 and MMDL 54702, since there are no photomicrographs and other information for these strains.

## 3. Discussion

### 3.1. Morphology Discussion

*S. seminulum* is similar to *S. terrestris* sp. nov. in valve width (3.0–4.5 µm in *S. seminulum* versus 3.7–6.1 µm in *S. terrestris* sp. nov.). The shape of the valves in the two species is different: *S. seminulum* has a linear-lanceolate shape, and *S. terrestris* sp. nov. has a linear-elliptical shape. The central area of *S. seminulum* is transversely widened and bow-tie-shaped; in *S. terrestris* sp. nov. the central area has an elliptical shape (Table 3). When *S. seminulum* was examined using SEM, the valves of the species have a smooth outer surface without grooves, both in the central area and at the apex of the valves [37]. In the case of *S. terrestris* sp. nov., there are longitudinal grooves in the central part of the valves (Figure 3M,N, Figure 4M,N and Figure 5M). The raphe in *S. seminulum* is not elevated above the valve surface; in *S. terrestris* sp. nov. the raphe lies in the conopeum (Figure 3M, Figure 4M and Figure 5M). We used the terms grooves and conopeum for the genus *Sellaphora,* following Mann et al. [8].

The grooves in *S. lundii* do not reach the ends of the valves, ending approximately at the first or second long striae in the central part of the valves [27]. *S. lundii* [27] has a evident sternum; likewise, in *S. terrestris* sp. nov., conopeum is present (Figure 3M,N, Figure 4M,N and Figure 5M). In addition, both species have a structural characteristic of some *Sellaphora*: round apical pits at the tops of the valve ends [27].

*S. laevissima* differs from our species in the greater width of valves (7–10 µm in *S. laevissima* versus 3.7–6.1 µm in *S. terrestris* sp. nov.). The striae density in *S. laevissima* is 15–21/10 µm, while in *S. terrestris* sp. nov. it is 28–35/10 µm. The areola density in S*. laevissima* is 30–35/10 µm, while in *S. terrestris* sp. nov. it is 60–65/10 µm. The central area of *S. laevissima* has the shape of a bow-tie, transversely expanded; in *S. terrestris* sp. nov. the central area has an elliptical shape (Table 3).

*S. balashovae* is similar in its length, width of valves, and density of areolae to *S. terrestris* sp. nov. (Table 3). In general, the density of areolae in *S. terrestris* sp. nov. is higher than that shown for *S. balashovae* (60–65/10 µm for *S. terrestris* sp. nov. versus 60/10 µm for *S. balashovae*). The central area of *S. balashovae* is transversely widened and in the shape of a bow-tie; in *S. terrestris* sp. nov., the central area has an elliptical shape (Table 3). The ultrastructural features of the species also allow them to be confidently distinguished. In *S. balashovae*, there are no grooves in the central area, in contrast to *S. terrestris* sp. nov. (Figure 3M,N, Figure 4M,N and Figure 5M). The raphe in *S. balashovae* lies in raised sternum, while in *S. terrestris* sp. nov., the raphe lies in a conopeum [11].

*S. davoutiana* Heudre, Wetzel, Moreau, and Ector differs from our species in the elliptical-lanceolate shape of the valves. In *S. terrestris* sp. nov. valve shape ranges from linear-elliptical to elliptical. The shape of the ends of the valves also differs between the two species: *S. davoutiana* has widely subcapitated, broadly rounded ends; while in *S. terrestris* sp. nov., the spices are short rostrate, to broadly rounded (Table 3). Valve widths, striae density, and areolae are similar across the species (Table 3). The central and axial areas of *S. davoutiana* are rather small; while in *S. terrestris* sp. nov. they are more visible. The central area of *S. davoutiana* can be either elliptical or rectangular; in *S. terrestris* sp. nov., the central area is elliptical.

Ultrastructurally, *S. davoutiana* does not have grooves in the axial and central area of the valves [39], which were shown for *S. terrestris* sp. nov.; the areolae of *S. davoutiana* are larger and more rectangular [39]; while in *S. terrestris* sp. nov., the areolae are not always rectangular and have a smaller diameter (Figure 3M, Figure 4M and Figure 5M). The raphe in *S. davoutiana* lies on an evident sternum [39].

*S. lundii* Wetzel, Barragán, and Ector [27] differs from *S. terrestris* sp. nov. by its elliptical shape of the valves (in *S. terrestris* sp. nov., the valves have a linear-elliptical shape) and wider ends of the valves (in *S. lundii*, the valves have a shape from rostrate to slightly capitate; in our species, the ends of the valves have a shape from rostrate to broadly rounded). The striae density in *S. lundii* is generally lower than in *S. terrestris* sp. nov. (23–28/10 µm versus 28–35/10 µm). The areolae of *S. lundii* are larger and rectangular, while the areole of *S. terrestris* sp. nov. are of a smaller diameter and only partially rectangular to mostly rounded. The main similarity between *S. lundii* and *S. terrestris* sp. nov. is the presence of distinct grooves in the central area of the valves on both sides of the proximal raphe ends.

When studying the morphological features of the new species, we noticed morphological similarity with some taxa from the genus *Microcostatus*. The genus *Microcostatus* was described in 1998 [44], primarily based on the presence of transversely oriented microcostae located in the axial area of the valve; the shape of the chloroplast, which is two plastids located along the cingulum of frustule [44]; and the lack of a conopeum but presence of an elevated sternum inside, with two longitudinal depressions alongside, sensu [44]. Initially, the type of genus (*M. krasskei*) showed open areolae and on this basis the authors assigned the genus to the family Naviculaceae [44]. Later, for representatives of the genus, a hymenes was shown that individually covered each of the areolae on the inner surface of the valves [41,43]. The diversity of morphological characters in the species currently assigned to the genus *Microcostatus* cannot but draw attention to itself. For example, the microcostae in the species can be expressed to a greater or lesser, the structure of the striae is different (they consist of one macroareola, for example, in the species *Microcostatus elisabethianus* Van de Vijver and Ector or several areolae in striae, as in most species of the genus), the conopeum/pseudoconopeum may be present or absent, and the hymenes closes the openings of the areolae in different species from the inside or even from the outside (*M. elisabethianus*). A common property for a number of species of *Sellaphora*, *Okhapkinia alexanderii* Glushchenko, Kulikovskiy, and Kociolek in Kulikovskiy et al. [5] and *Microcostatus* (e.g., *M. dexteri*, *M. egregius*, *M. naumannii*) is the presence of a round apical pit on each internal valve margin.

*Sellaphora terrestris* sp. nov. is morphologically similar to *Microcostatus dexterii* Stanek-Tarkowska, M. Rybak, and Czyż [43] and *Microcostatus naumanii* (Hustedt) Lange-Bertalot [41] (Table 3). The greatest similarities between these species are observed using SEM. *Microcostatus dexterii* [43] and *S. terrestris* sp. nov. (Figure 3M–O, Figure 4M,N and Figure 5M) have a raised, slightly asymmetrical raphe sternum. The orientation of the outer central and distal raphe ends also coincides in the species. As in the case of *S. terrestris* sp. nov. (Figure 3M,N, Figure 4M,N and Figure 5M), *M. dexterii* has longitudinal depressions that widen into the valve center and form an almost longitudinal lanceolate central area [43]. The authors did not indicate any pronounced microcostae, which are important morphological feature of the genus *Microcostatus*. Both species have a structure typical for some *Sellaphora*, that is, round apical pits at the apex of the valve ends [43]. *Microcostatus dexterii* differs from *S. terrestris* sp. nov. by its valve shape (lanceolate to elliptical-lanceolate vs linear-elliptical to elliptical), lower striae density (20–24/10 µm vs 28–35/10 µm), and areolae (45–50/10 µm vs 60–65/10 µm). The shape of the areolae in *M. dexterii* is also almost square, in contrast to *S. terrestris* sp. nov., where the shape of the areolae can be either almost square or rounded. The ends of the valves of the species *M. dexterii* are widely rounded; in *S. terrestris* sp. nov., the shape of the ends is more varied, from rostrate to widely rounded.

*Microcostatus naumanii* is similar to *S. terrestris* sp. nov. in its valve width (4–5 µm in *M. naumanii* vs 3.7–6.1 in *S. terrestris* sp. nov.). The main similarity of the discussed species was observed in the SEM. The areolae of both species are round to rectangular in shape. In both species, the raphe is located on a raised and slightly asymmetrical sternum; the orientation of the outer central and proximal raphe ends of the raphe is the same [41] (pp. 146–420), (Figure 3M,P,Q, Figure 4M,P and Figure 5M–O). Both species, *M. naumanii* [41] and *S. terrestris* sp. nov. (Figure 3M,N, Figure 4M,N and Figure 5M), have prominent longitudinal depressions widening towards the central part of the valves; the central area in both species is longitudinally lanceolate [41], (Figure 3N and Figure 4N). In addition, *M. dexterii*, some *Sellaphora* spp. (including *S. terrestris* sp. nov.), *Okhapkinia alexanderi*, and *M. naumanii* have one common structure: round apical pits at the apices of the valve ends [41] (Figure 3Q and Figure 4P,R). Van de Vijver et al. [41] indicated the presence of very weakly expressed microcostae in the material of *M. naumanii* [41]; while in Zidarova et al. [42], microcostae were not present in the same species.

### 3.2. Molecular Discussion

Molecular genetic data on freshwater representatives of *Microcostatus* are not available. At present, the 18S rRNA gene sequences of two strains of *Microcostatus* (MMDL 54701, MMDL 54702) from the intertidal zone of China have been deposited in GenBank. Unfortunately, the work has not yet been published, and there is no way to assess the morphological features of these taxa. Furthermore, to date, *Microcostatus* has been known only as a freshwater taxon, raising questions as to the identity of the diatoms from which the Chinese sequence data have been obtained. Despite this, the results of calculating the genetic distance for the site at V4 (Table 2) showed a high degree of difference. Thus, the sequences of strains differ from each other by 6.9% and most likely represent different species. In relation to the species *S. terrestris* sp. nov., the differences were 4.6% and 8.2%, respectively, which clearly demonstrates their isolated position. However, to understand the relationship between *Microcostatus* and *Sellaphora*, a phylogenetic analysis is required involving an additional set of genes (for example, *rbc*L, *cox*1), since the 18S rRNA analysis did not provide the necessary resolution. For accurate identification, it is necessary to study the morphology of these strains. Both genera, as currently circumscribed, possess a tremendous amount of morphological variation. For example, Liu et al. [45] recognized four groups differing in their morphological attributes, and one of them (their group B) is mainly represented by taxa in molecular analyses. All this, in our opinion, indicates the need to revise both genera: *Microcostatus* and *Sellaphora*. Given that the monophyletic clade containing “*S. seminulum*” was distinct from other *Sellaphora* species in both analyses, it may be prudent to reinvestigate whether the genus *Eolimna* should be recognized as distinct.

Studies using molecular approaches indicate a high genetic diversity of *Sellaphora*. Using the example of *S. pupula*, a number of studies have shown groups of representatives with minor morphometric differences form separate well-maintained genetic lines [6,9,14]. The results of crossing some taxa confirmed the existence of reproductive barriers for morphologically similar populations [13,19]. In studies concerning the detailed study of the morphological and cryptic diversity of *Sellaphora*, many representatives with unclear differences in morphology and with pronounced genetic isolation were considered as phenodemes or demes [6,9,14]. A detailed revision of the morphological, molecular, and crossbreeding data was carried out using the example of four species of *S. americana* (Ehrenberg) D.G. Mann, *S. bacillum*, *S. pupula*, and *S. laevissima* sensu Krammer and Lange-Bertalot [24] from 40 sites in Britain and Northern Ireland by Mann et al. [8]. As a result, 54 demes were proposed, which can be considered the starting point for further research into the intraspecific and interpopulation relationships of *Sellaphora*.

Molecular data are most fully summarized in Mann and Poulíčková [12], where a phylogenetic analysis of 67 strains was carried out based on a concatenated five-gene dataset (partial nuclear 18S rDNA and 28S rDNA, plastid 23S rDNA and *rbc*L, and mitochondrial *cox*1). The authors noted that the tree topology supported the results obtained in previous works based on a two-gene dataset [9] (18S rDNA and *rbc*L); [20] (*cox*1 and *rbc*L)) except for *S.* cf. *minima* (clone BM42), whose position in early studies was inside the *S. pupula*–*S. bacillum* complex. In the latest phylogeny of *S. minima* (TCC 524) and *S.* cf. *minima* (BM42), these strains form a separate clade next to the clade including *S. laevissima* (Figure 5) [12]. The results of the phylogenetic analysis by Mann and Poulíčková (Figure 5) [12] indicated that most strains are grouped into three clades named 1, 2, and 3: clades 1 and 3 include the taxa *S. pupula* sensu lato, and clade 2 includes strains of *S. bacillum* and several demes of *S. pupula*, characterized by the presence of conopeum (continuous across the central area or interrupted accordingly), while representatives of *S. laevissima*, *S. seminulum*, and *S. minima* are grouped into separate external branches.

Our phylogenetic analysis for the 18S rDNA and *rbc*L genes supports the clades 1–3 identified in previous works [9,12]. However, the clade size is much smaller, due to the lack of 18S rDNA gene sequence data for many strains. The outer clades “*minima*”, “*seminulum*”, and “*laevissima*” are also clearly differentiated with sufficient support values (Figure 7). It should be noted that the identification of these external clades was also observed in previous works. Thus, the clade with *S. minima* (strains BM42, TCC524) had the highest support (1.0 PP) in Mann and Poulíčková [12], the clade with *S. laevissima* (indicated as clade 4 in Evans et al. [9]; Poulíčková et al. [21]), regardless of the genes used, had a stable isolated phylogenetic position: 1.0 PP for 18S rDNA and *rbc*L analyses [8], 85 LB for *cox*1 and *rbc*L analyses [20], 0.99 PP for five-gene dataset (18S rDNA, 28S rDNA, 23S rDNA, *rbc*L, *cox*1) [11]. In our analysis, this clade is supplemented by *S. balashovae*.

A high level of support for the clade with *S. seminulum* was first shown by Mann and Poulíčková [12], where three strains were included in the analysis. In previous works [9,21], only one strain (TM37) was included, presumably assigned to this species. Notably, in all analyses, this species always represented a separate line (branch) next to *S. laevissima* (clade 4).

We performed a more complete phylogenetic analysis for the *rbc*L region, since the number of sequences in GenBank for this gene for *Sellaphora* is much larger. Our phylogenetic tree includes 106 strains of *Sellaphora*, the taxonomic epithets listed in GenBank have been changed to the names of demes or species proposed in the relevant works (listed in Supplementary Data S1). The general topology of the tree is consistent with that of previous works [9,12,20,21]. To designate subclades, we have used the numbers proposed by Evans and co-authors [9] and Mann and Poulíčková [12]. Clade 1A includes six demes of *S. auldreekie*, clade 1B of *S. bisexualis*, and several demes of *S. pupula*. Clade 2 in our phylogeny is expanded, due to a separate subclade, which includes six demes of *S. pupula*. Clade 3 retains high statistical support, includes many *S. pupula* demes, and strains of *S. blackfordensis* D.G. Mann and S. Droop and *S. capitata* are grouped into subclades. The fourth clade is formed by strains of *S. laevissima* and *S. balashovae*. The new basal clade has maximum support, which includes two clades: *S. seminulum* and a clade consisting of strains of the species *S. terrestris* sp. nov. The clade with *S. minima* was supplemented by the strain *Sellaphora* sp. Ak1876, recently isolated at Lake Toro, Sibecya, Hokkaido, Japan [46]. In general, according to the molecular genetic data of *Sellaphora terrestris* sp. nov., it was most similar to the species *S. seminulum*, *S. laevissima,* and *S. balashovae* (Figure 6 and Figure 7).

Thus, the morphological data and molecular genetic data indicate that our species belongs to the genus *Sellaphora*. The genus *Microcostatus* can currently be considered to require additional morphological and molecular genetic study of the species currently assigned to this genus.

## 4. Materials and Methods

Soil samples were collected from Cát Tiên National Park, Đồng Nai Province, Vietnam, by E.S. Gusev and E.M. Kezlya in June 2019. Sampling occurred during an expedition of the Joint Russian-Vietnamese Tropical Research and Technological Centre (the “Ecolan 3.2” Project). The National Park is located 150 km northeast of Hồ Chí Minh City. The region belongs to the bioclimatic type of monsoon tropical climate with summer rains, relative humidity almost always exceeding 70%, and average annual temperature of about 26 °C. The main part of the territory is occupied by forests, which are of the monsoon, semi-deciduous type [47].

### 4.1. Sample Collection Procedure

The samples were collected as follows: the surface of the test site was examined in order to detect macrogrowth of algae, then a composite sample was taken from an area of 10–30 m^2^. The composite sample consisted of 5–10 individual samples. For an individual sample, the topsoil was removed from an area of 5 to 20 cm^2^ with a metal scoop or shovel. After sampling, the instruments were cleaned and sterilized with ethanol. The samples were placed in labeled plastic zip bags and carried to the laboratory. The absolute humidity was measured immediately using the hot-drying method [48], and the samples were air-dried and packaged.

### 4.2. Soil Acidity Measurements

To measure pH, we mixed 30 g of soil with 150 mL of distilled water [49]. The suspension was poured into a clean glass beaker, and the measurements were performed with a Hanna Combo (HI 98129) device (Hanna Instruments, Inc., Woonsocket, RI, USA).

### 4.3. Culturing

Gathered materials were processed in the laboratory of molecular systematics of aquatic plants of the Institute of Plant Physiology of the Russian Academy of Sciences (IPP RAS). A sample of soil was thoroughly mixed and a small amount (15–20 g) was placed into a Petri dish (diameter 60 mm), then saturated with distilled water up to 60–80% of full moisture capacity and placed into an illuminated climate chamber. After a 10-day incubation period, the sample was diluted with a small amount of distilled water, mixed gently, and the suspension was transferred to a Petri dish for LM, using a Zeiss Axio Vert A1 inverted microscope. Algal cells were extracted with a micropipette, washed in 3–5 drops of sterile distilled water, and placed into a 300-µL well on a plate for enzyme-linked immunoassay with Waris-H+Si [50]. Non-axenic unialgal cultures were maintained at 22–25 °C in a growth chamber with a 12:12 h light:dark photoperiod. After 20 days, strains were re-inoculated to a Petri dish with liquid Waris-H+Si medium. The strains investigated here were designated VP272, VP299, VP303, and VP304 (see Table 4).

A list of all samples and strains examined in this study, with their GenBank accession numbers and geographic location of sampling sites, with measured ecological parameters is presented in Table 4. Terminology of the valve follows Kulikovskiy et al. [4], Mann et al. [8], Wetzel et al. [37], Van de Vijver et al. [41], and Stanek-Tarkowska et al. [43].

### 4.4. Preparation of Slides and Microscope Investigation

The monoclonal culture was boiled in 30% hydrogen peroxide at a temperature between 150–160 °C for 8 h, to dissolve organic matter. After decanting and refilling up to 50 mL with deionized water, the suspension was spread onto coverslips and left to dry at room temperature. Permanent diatom preparations were mounted in Naphrax^®^ (refraction index = 1.73). LM observations were performed with a Zeiss Axio Scope A1 microscope equipped with an oil immersion objective (×100, n.a. 1.4, differential interference contrast) and Axio Cam ERc 5s camera. Valve ultrastructure was examined in Papanin Institute for Biology of Inland Waters RAS, Borok, Russia, with a JSM-6510LV scanning electron microscope (JEOL Ltd., Tokyo, Japan), operated at 10 kV and an 11-mm distance. For scanning electron microscopy (SEM), parts of the suspensions were fixed on aluminum stubs after air-drying. The stubs were sputter coated with 50 nm of gold in an Eiko IB 3 (Eiko Engineering Co., Ltd, Ibaraki, Japan).

### 4.5. Molecular Investigation

The total DNA of the strains was obtained using ChelexTM 100 Chelating Resin, molecular biology grade (Bio-Rad Laboratories, Hercules, CA, USA), according to the manufacturer’s protocol 2.2. Fragments of 18S rDNA gene (406–417 bp, including V4 domain), and partial *rbc*L plastid gene (951–957 bp) were amplified using the primers D512for and D978rev from Zimmerman et al. [51] for 18S rDNA fragments, and *rbc*L40+ from Ruck and Theriot [52] and *rbc*L1255 from Alverson et al. [53] for the *rbc*L gene. Amplifications of the 18S rDNA fragments and partial *rbc*L gene were carried out using the premade mix, ScreenMix (Evrogen, Moscow, Russia), for polymerase chain reaction (PCR).

The conditions of amplification for 18S rDNA fragments were as follows: an initial denaturation of 5 min at 95 °C, followed by 35 cycles at 94 °C for denaturation (30 s), 52 °C for annealing (30 s) and 72 °C for extension (50 s), and a final extension of 10 min at 72 °C. The conditions of amplification for partial *rbc*L were an initial denaturation of 5 min at 95 °C, followed by 45 cycles at 94 °C for denaturation (30 s), 59 °C for annealing (30 s) and 72 °C for extension (80 s), and a final extension of 10 min at 72 °C.

The resulting amplicons were visualized using horizontal agarose gel electrophoresis (1.5%), colored with SYBR Safe (Life Technologies, Carlsbad, CA, USA). Purification of DNA fragments was performed with an ExoSAP-IT kit (Affimetrix, Santa Clara, CA, USA), according to the manufacturer’s protocol. 18S rDNA fragments and partial *rbc*L gene were decoded from two sides, using forward and reverse PCR primers and the Big Dye system (Applied Biosystems, Waltham, MA, USA), followed by electrophoresis using a Genetic Analyzer 3500 sequencer (Applied Biosystems, Waltham, MA, USA).

Editing and assembly of the consensus sequences were carried out by comparing the direct and reverse chromatograms using the Ridom TraceEdit program (ver. 1.1.0) and Mega7 [54].

With Mega7 software, the p-distance for 18S rDNA gene was determined and used to calculate the sequence similarity with the formula (1–p) ×100. Newly determined sequences and 18S rDNA gene and *rbc*L fragments from 39 other diatoms, which were downloaded from GenBank (taxa and Accession Numbers are given in the Supplementary Data S1), were used to generate two-gene trees (Supplementary Data S2). Since the 18S rDNA gene sequences are not available for the most *Sellaphora* strains, the *rbc*L reads were included in the alignments, along with corresponding sequences of 110 diatoms downloaded from GenBank (Supplementary Data S3). Three centric diatom species were chosen as the outgroups. The nucleotide sequences of the 18S rDNA and *rbc*L genes were aligned separately using Mafft v7 software and the E-INS-i model [55]. For the protein-coding sequences of the *rbc*L gene, we checked that the beginning of the aligned matrix corresponded to the first position of the codon (triplet). The resulting alignments had lengths of 404 (18S rDNA) and 957 (*rbc*L) characters. Two 18S rDNA gene sequences of the genus *Microcostatus* were used for the p-distance calculation.

The dataset was analyzed using the Bayesian interference (BI) method implemented in Beast ver.1.10.1 [56] for phylogeny reconstruction. For each of the alignment partitions, the most appropriate substitution model was estimated using the Bayesian information criterion (BIC), as implemented in jModelTest 2.1.10 [57]. This BIC-based model selection procedure selected the following models, shape parameter α, and proportion of invariable sites (pinvar) for the concatenated tree: TIM3ef+I+G, α = 0.3800, and pinvar = 0.5940 for 18S rDNA gene; HKY+I, pinvar = 0.8430 for the first codon position of the *rbc*L gene; TVMef+I, pinvar = 0.9000 for the second codon position of the *rbc*L gene; and TPM3uf+G, α = 0.4070 for the third codon position of the *rbc*L gene. We used the HKY model of nucleotide substitution instead of TIM3ef and TPM3uf, and the GTR model instead of TVMef, since they were the best matching models available for BI. The following models, shape parameter α, and proportion of invariable sites were for the *rbc*L tree: HKY+I+G, α = 0.3370, and pinvar = 0.6700 for the first codon position of the *rbc*L gene; TrNef+I+G, α = 0.5340, and pinvar = 0.8280 for the second codon position of the *rbc*L gene; and TVM+I+G, α = 0.6170, and pinvar = 0.2080 for the third codon position of the *rbc*L gene. We used the HKY model of nucleotide substitution instead of TrNef, and the GTR model instead of TVM, for the individual *rbc*L tree.

A Yule process tree prior was used as a speciation model. The analysis ran for 5 million generations, with chain sampling every 100 generations. The parameters estimated convergence, effective sample size (ESS), and burn-in period were checked using the software Tracer ver. 1.7.1. [56]. The initial 25% of the trees were removed, and the rest were retqined to reconstruct a final phylogeny. The phylogenetic tree and posterior probabilities of its branching were obtained on the basis of the remaining trees, having stable estimates of the parameter models of nucleotide substitutions and likelihood. Maximum likelihood (ML) analysis was performed using the program RA×ML [58]. The nonparametric bootstrap analysis with 1000 replicates was used. The phylogenetic tree topologies are available online: Supplementary Data S4 for the concatenated *rbc*L and 18S rDNA genes tree and Supplementary Data S5 for the *rbc*L gene tree. The statistical support values were visualized in FigTree (ver. 1.4.2) and Adobe Photoshop CC (19.0).

## 5. Conclusions

We studied in detail the morphology of four soil strains of *Sellaphora* from Cát Tiên National Park and identified a number of differences from all currently known species. During the study of cultured and natural materials, variability of morphometric characteristics (length and width of valves, density of striae) was revealed, while molecular studies on *rbc*L and 18S rDNA genes including the V4 domain showed that the strains studied by us formed a separate branch, with high statistical support. Based on these data, we described the new for science species *Sellaphora terrestris* sp. nov. In addition, we noticed that some species assigned to *Microcostatus* J.R. Johansen and J.C. Sray have a very similar morphology to our new species. The results of the study of the morphological characteristics of a number of species of *Sellaphora* and *Microcostatus* indicated that many characteristics (robustness of microcostae, striae morphology, and presence of conopeum and hymenes) can be expressed to varying degrees. We cannot exclude the possibility that some species assigned to the genus *Microcostatus* do not belong there, and may be better placed within *Sellaphora*. Molecular genetic data for *Microcostatus* are limited and require further study.

## Figures and Tables

**Table 1:** Comparison of morphological characteristics of wild populations and cultures of *S. terrestris* sp. nov.

	Wild Population			Culture			
	Sample Kt 33	Sample Kt 59	Sample Kt 60	Strain VP 272	Strain VP 299	Strain VP 303	Strain VP 304
Outline	linear-lanceolate, linear-elliptical to elliptical	linear-lanceolate, linear-elliptical to elliptical	linear-elliptical to elliptical	linear-elliptical to elliptical	elliptical	linear-elliptical	linear-elliptical
Ends	short rostrate to broadly rounded	short rostrate to broadly rounded	short rostrate to broadly rounded	rostrate	short rostrate to broadly rounded	rostrate	rostrate
Axial area	narrow	narrow	narrow	narrow	narrow	narrow	narrow
Central area	small, elliptical	small, elliptical	small, elliptical	small, elliptical	small, elliptical	small, elliptical	small, elliptical
Valve length (μm)	9.1–17.1 (11.7 ± 2.4; n = 22)	8.2–16.9 (12.3 ± 2.3; n = 22)	6.9–13.4 (9.5 ± 1.4; n = 22)	10.4–13.9 (11.7 ± 0.8; n = 22)	5.2–6.3 (5.5 ± 0.2; n = 21)	13.2–14.0 (13.5 ± 0.2; n = 23)	13.4–14.3 (13.8 ± 0.2; n = 24)
Valve width (μm)	4.5–6.1 (4.9 ± 0.4; n = 22)	4.2–5.0 (4.5 ± 0.2; n = 22)	4.3–5.0 (4.4 ± 0.2; n = 22)	4.5–4.9 (4.7 ± 0.1; n = 22)	3.7–4.0 (3.8 ± 0.05; n = 21)	4.7–4.9 (4.8 ± 0.03; n = 23)	4.6–5.1 (4.8 ± 0.1; n = 24)
Striae, in 10 μm	29–32 (28.6 ± 1.3; n = 22)	30–31 (30.1 ± 0.3; n = 22)	29–31 (29.9 ± 0.4; n = 22)	29–30 (29.9 ± 0.2; n = 22)	30–34 (32 ± 0.7; n = 21)	28–32 (30 ± 0.6; n = 23)	30–35 (32.1 ± 0.9; n = 24)
Areolae	-	-	-	-	ca. 65	ca. 60	ca. 60

**Table 2:** Percentage similarity (p-distance) matrix of 20 strains on the basis of the partial 18S rRNA gene (401 bp).

	Strain	1	2	3	4	5	6	7	8	9	10	11	12	13	14	15	16	17	18	19	20
**1**	*Sellaphora terrestris* VP304	–																			
**2**	*Microcostatus* sp. MMDL 54702	95.4	–																		
**3**	*Microcostatus* sp. MMDL 54701	91.8	92.9	–																	
**4**	*Sellaphora auldreekie* DUN1 clade_1A	96.4	94.4	90.8	–																
**5**	*Sellaphora pupula agg.* “large” THR11 clade 1B	95.7	95.2	90.8	97.7	–															
**6**	*Sellaphora bisexualis* RBG1 clade 1B	95.9	95.4	91.1	98.0	99.8	–														
**7**	*Sellaphora lanceolata* BLA13 clade 2A	96.4	94.4	90.0	97.4	96.7	96.9	–													
**8**	*Sellaphora bacillum* BLA3 clade 2A	96.7	95.2	90.8	97.7	97.0	97.2	99.2	–												
**9**	*Sellaphora pupula agg.* “little” RBG clade 2B	95.4	95.4	91.1	97.5	99.5	99.8	96.4	96.7	–											
**10**	*Sellaphora pupula agg.* “small capitate” DUN7 clade 2B	95.9	95.4	91.1	98.0	99.8	99.7	97.2	97.5	99.5	–										
**11**	*Sellaphora blackfordensis* BLA6 clade 3	95.9	95.1	90.8	97.9	97.2	97.4	97.2	97.7	96.9	97.4	–									
**12**	*Sellaphora pupula agg.* cf. *obesa* BEL2 clade 3	95.4	94.9	90.5	97.2	96.9	97.2	96.4	96.9	96.7	97.2	99.2	–								
**13**	*Sellaphora balashovae* SvA-2018a B385	96.7	95.7	91.3	96.9	96.4	96.7	96.7	97.2	96.2	96.7	96.9	96.2	–							
**14**	*Sellaphora* sp. KEL-2015 JAR35 4FRun6	95.9	94.6	90.3	97.4	96.9	97.2	96.7	97.2	96.7	97.2	98.5	97.7	96.9	–						
**15**	*Sellaphora minima* TCC524	96.2	93.9	90.8	95.9	95.2	95.4	95.4	95.7	95.4	95.4	94.9	94.1	95.7	94.9	–					
**16**	*Sellaphora laevissima* THR4	97.2	96.7	91.8	96.9	97.2	97.4	97.2	97.7	96.9	97.4	98.0	97.4	98.5	98.0	95.9	–				
**17**	*Sellaphora laevissima* THR1	96.4	95.9	91.1	96.2	96.4	96.7	96.4	96.9	96.2	96.7	97.2	96.7	97.7	97.2	94.9	99.2	–			
**18**	*Sellaphora seminulum* HYU-D014	94.9	92.6	89.0	95.2	94.4	94.6	95.9	95.9	94.2	94.9	94.9	94.2	94.9	93.9	93.7	94.9	94.7	–		
**19**	*Sellaphora seminulum* TCC461	94.1	93.4	89.0	93.6	93.9	94.1	93.6	93.6	93.6	94.4	94.1	93.9	94.1	93.6	93.9	95.4	94.6	95.7	–	
**20**	*Sellaphora* cf. *seminulum* TM37	95.9	94.4	90.8	96.7	96.2	96.4	96.7	97.0	96.4	96.4	96.5	95.7	96.9	95.4	95.4	96.4	95.7	96.7	94.7	–

**Table 3:** Comparison of *Sellaphora terrestris* sp. nov. with similar species of *Sellaphor*a and *Microcostatus*.

	Outline	Ends	Axial Area	Central Area	Valve Length (μm)	Valve Width (μm)	Striae in 10 μm	Areolae in 10 μm	Distribution	References
*Sellaphora* *terrestris* sp. nov.	linear-elliptical to elliptical with weakly convex margins	short rostrate to broadly rounded	narrow, weakly extended to the central area	small, longitudinally lanceolate	6.9–17.1	3.7–6.1	28–35	60–65	Vietnam, Cát Tiên National Park	This study
*S. laevissima*	linear, usually with weakly convex margins in the centre	either very weakly protracted, subcapitate or not protracted, always broadly rounded	narrow, not or very little broadened towards the centre	distinct, more or less transversely rectangular, extends over 50–60% of the valve width, rarely more	22–50	7–11	15–21	30–35	Holarctic	[38]
*S. balashovae*	elliptic	subcapitate	narrow, weakly extended to the central area	bow-tie-shaped, bordered by alternating short and long striae	11–14	4	30	60	Russia, Lake Frolikha	[11]
*S. seminulum*	linear–lanceolate to broadly lanceolate with clearly convex margins (inflated in the middle)	protracted, broadly rounded, slightly rostrate	linear–lanceolate, with an irregular border, and is central or apparently very slightly displaced towards the secondary side	bordered by three shorter striae and is usually butterfly–shaped, but the size and shape vary	5.0–16.5	3.0–4.5	18–20	>30	Holarctic	[37,38]
*S. davoutiana*	elliptic-lanceolate	broadly protracted, subcapitate and rounded	narrow, linear and slightly widened toward central area	small and elliptical to rectangular with 2 or 3 slightly shortened striae of varying length on either side	28–35	4.0–4.9	26–30	60–70	France. Grand Est region. La Bresse: Lispach Lake	[39]
*S. lundii*	elliptical, are centrally inflated	rostrate to weakly capitate	narrow, linear	clearly marked, rectangular to oblong	9–13	4–5	23–28	55–60 *	England, UK, Selly Oak, Birmingham. Allotment; Belgium, Luxembourg	[27,40]
*Microcostatus naumanii*	elliptic-lanceolate to lanceolate with clearly convex margins	in smaller specimens, apices tend to be more rostrate, whereas larger valves have protracted, capitate ends	very narrow near the valve apices but forming two longitudinal depressions, abruptly and elliptically enlarged towards the central area	almost elliptical	9.5–15.5	4–5	24–26	c.a. 60 *	Sweden, Macedonia, Norway, Finland, Victoria Island, Scotland, Patagonia (Argentina), South Shetland Islands (Deception Island, King George Island and Livingston Island), Antarctic Continent	[41,42]
*Microcostatus dexterii*	lanceolate to elliptical-lanceolate	rounded	narrowing near the valve apices, forming two longitudinal depressions, enlarged in the middle and creating an central area	almost lanceolate	6.6–16.4	2.3–5.5	20–24	45–50 *	Poland. Podkarpacie Province, Rzeszów, soils	[43]

* counted by us from published data.

**Table 4:** List of strains examined in this study, with their GenBank accession numbers. Geographic locality of samples and measured ecological parameters are indicated.

Strains	Slide No	Sample No	Sample Locality	Collection of Date	Collection Site	pH	Total Humidity (%)	GenBank Accession Number, SSU rDNA, Partial	GenBank Accession Number, *rbc*L, Partial
*Sellaphora terrestris* sp. nov. VP 272	07030	Kt33	Southeast Vietnam, Cat Tien National Park	25 June 2012	11°25.019′ N 107°25.545′ E Forest soil surface	6.0	48.97	ON332054	ON350763
*Sellaphora terrestris* sp. nov. VP 299	07057	Kt60	Southeast Vietnam, Cat Tien National Park	25 June 2019	11°24.247′ N 107°23.661′ E Agricultural field soil surface	4.58	27.9	ON332055	ON350764
*Sellaphora terrestris* sp. nov. VP 303	07061	Kt59	Southeast Vietnam, Cat Tien National Park	25 June 2019	11°24.241′ N 107°22.468′ E Agricultural field soil surface	5.22	36	ON332056	ON350765
*Sellaphora terrestris* sp. nov. VP 304	07062a	Kt59	Southeast Vietnam, Cat Tien National Park	25 June 2019	11°24.241′ N 107°22.468′ E Agricultural field soil surface	5.22	36	ON332057	ON350766

## Data Availability

Not applicable.

## Supplementary Materials

The following supporting information can be downloaded at: https://www.mdpi.com/article/10.3390/plants11162148/s1, Data S1: Taxa and DNA sequence data used in phylogenetic analysis (.docx format), Data S2: Alignment file of 43 concatenated *rbc*L, 18S rDNA genes sequences of Sellaphoraceae (.fas format), Data S3: Alignment file of 114 *rbc*L gene sequences of Sellaphoraceae (.fas format), Data S4: The topology of the concatenated *rbc*L, 18S rDNA genes Bayesian phylogenetic tree (.txt format), Data S5: The topology of the *rbc*L gene Bayesian phylogenetic tree (.txt format).
