# Supplementary material for: Description of the Soil Diatom Sellaphora terrestris sp. nov. (Bacillariophyceae, Sellaphoraceae) from Vietnam, with Remarks on the Phylogeny and Taxonomy of Sellaphora and Systematic Position of Microcostatus
Source: Plants (Basel). 2022 Aug 18;11(16):2148. doi: 10.3390/plants11162148 (PMC9415725; doi:10.3390/plants11162148)
Supplement: Supplementary file 1 [file plants-11-02148-s001.zip › Supplementary Data S1 new.pdf]

Supplementary table S1. Taxa and DNA sequence data used in phylogenetic analysis.

Clade	Strain	taxa according to GenBank	taxa or provisional names according to references	Origin (Date)	the GenBank accession no.	
					*rbc*L	18S rDNA
Clade 1A	Sel781INV	*S. auldreekie*	*S. auldreekie*	United Kingdom: 55.9614 N 3.0679 W (2008)	KC911805	–
	DUN3	*S. auldreekie*	*S. auldreekie*	United Kingdom: Scotland, Edinburgh, Dunsapie Loch 55.94 N 3.15 W (2003)	EF143277	–
	DUN2	*S. auldreekie*	*S. auldreekie*	Scotland: Dunsapie Loch (2003)	EF143268	–
	Sel776INV	*S. auldreekie*	*S. auldreekie*	United Kingdom: 55.9614 N 3.0679 W (2007)	KC911804	–
	BLA2	*S. auldreekie*	*S. auldreekie*	United Kingdom: Scotland, Edinburgh, Blackford Pond 55.92 N 3.20 W (2004)	EF143303	–
	DUN4	*S. auldreekie*	*S. auldreekie*	Scotland: Dunsapie Loch (2003)	EF143267	–
	BLA1	*S. auldreekie*	*S. auldreekie*	Scotland: Blackford Pond NT 253709 (1996)	EF143320	–
Clade 1A	Cr44	*S. auldreekie*	*S. auldreekie*	Australia: 37.8003 S 145.2819 E (2007)	KC911803	–
	DUN1	*S. auldreekie*	*S. auldreekie*	Scotland: Dunsapie Loch NT 281731 (1996)	EF143276	EF151965
	mm185	*S. pupula*	*S. pupula* agg. "mumblin auldreekie"	Australia: 38.3189 S 142.9144 E (2007)	KC911816	–
	mm135	*S. pupula*	*S. pupula* agg. "mumblin auldreekie"	Australia: 38.3189 S 142.9144 E (2007)	KC911814	–
	mm178	*S. pupula*	*S. pupula* agg. "mumblin auldreekie"	Australia: 38.3189 S 142.9144 E (2007)	KC911815	–
	Sel629K	*S. pupula*	*S. pupula* agg. "southern auldreekie"	Australia: 37.7881 S 145.0433 E (2007)	KC911807	–
	RBGE Kew21	*S. pupula*	*S. pupula* agg. "southern auldreekie"	Australia: 37.7881 S, 145.0433 E (2007)	HQ317119 HQ337598	–
	KewA3	*S. pupula*	*S. pupula* agg. "southern auldreekie"	Australia: 37.7881 S 145.0433 E (2007)	KC911806	–

	Str13	*S. pupula*	*S. pupula* agg. “southern auldreekie”	Australia: 37.7286 S 145.0944 E (2007)	KC911811	–
	BB94	*S. pupula*	*S. pupula* agg. “another auldreekie”	Australia: 37.77 S 145.08 E (2007)	HQ317121<br>HQ337600	–
	BolinA2	*S. pupula*	*S. pupula* agg. “coarse auldreekie”	Australia: 37.7692 S 145.0783 E (2007)	KC911808	–
	Sel627K	*S. pupula*	*S. pupula* agg. “coarse auldreekie”	Australia: 37.7881 S 145.0433 E (2007)	KC911810	–
	Sel642K	*S. pupula*	*S. pupula* agg. “coarse auldreekie”	Australia: 37.7881 S 145.0433 E (2007)	KC911809	–
	LE J9	*S. pupula*	*S. pupula* agg. “Czech auldreekie”	United Kingdom: 55.9606 S 3.1618 E (2007)	KC911812	–
Clade 1A	LE D35	*S. pupula*	*S. pupula* agg. “Czech auldreekie”	United Kingdom: 55.9606 S 3.1618 E (2007)	KC911813	–
	RGBE 28/28	*S. pupula*	*S. pupula* agg. “Czech auldreekie”	Czech Republic: Cerny pond 50.61 N 14.76 E	HQ317129<br>HQ337609	–
	Bfp04	*S. blackfordensis*	*S. blackfordensis*	No date	JN418674	–
	KME 2009a	*S.* sp.	*S. bisexualis* SEL9B	United Kingdom: Scotland, Edinburgh, Blackford Pond 55.92 N 3.20 W (2003)	HQ317111<br>HQ337609	–
	RBG1	*S. pupula*	*S. pupula* agg. “elliptical”<br>*S. bisexualis*	Scotland: Royal Botanic Garden Pond (NT 248753)	EF143271	EF151962
Clade 1B	BLA15	*S. pupula*	*S. pupula* agg.“elliptical”	Scotland: Blackford Pond (2003)	EF143321	–
	BLA14	*S. pupula*	*S. pupula* agg. “elliptical”<br>*S. bisexualis*	Scotland: Blackford Pond (2003)	EF143294	–
	THR9	*S. pupula*	*S. pupula* agg. “elliptical”<br>*S. pupula* agg. “upland elliptical”	United Kingdom: Scotland, Threipmuir Reservoir 55.86 N 3.33 W (2003)	EF143297	EF151972

	THR10	*S. pupula*	*S. pupula* agg. “elliptical”<br>*S. pupula* agg. “upland elliptical”	Scotland: Threipmuir Reservoir (2003)	EF143296	–
Clade 1B	RGBE B155	*S. pupula*	*S. pupula* agg. “southern elliptical”	Australia: Victoria, Grampians, Lake Bellfield 37.18 S 142.54 E(2007)	HQ317120 HQ337599	–
	THR13	*S. pupula*	*S. pupula* agg. “small blunt–capitate”	Scotland: Threipmuir Reservoir (2003)	EF143272	–
	THR8	*S. pupula*	*S. pupula* agg. cf. “small blunt–capitate”	Scotland: Threipmuir Reservoir (2003)	EF143308	–
	GER1	*S. pupula*	*S. pupula* agg. “europa”	Germany: Friedheimer See 53°31′23″ N, 8°39′54″ E (1997)	EF143318	EF151984
	THR11	*S. pupula*	*S. pupula* agg. “large”	Scotland: Threipmuir Reservoir (1997)	EF143285	EF151970
Clade 2A	BLA5	*S. bacillum*	*S. bacillum*	Scotland: Blackford Pond (2003)	EF143284	–
	BLA4	*S. bacillum*	*S. bacillum*	Scotland: Blackford Pond (2003)	EF143311	–
	E3147	*S. bacillum*	*S. bacillum* BLA3	Scotland: Blackford Pond (1996)	AY571745	EF151980
	ST-MAR1	*S. bacillum*	*S. bacillum*	Scotland: St Margarets Loch (1996)	EF143273	–
	SEL601As	*S. obesa*	*S. obesa*	United Kingdom: England, Ashford lake 53.23 N 1.70 W (2007)	HQ337592	–
	THR14	*S. pupula*	*S. pupula* agg. “small lanceolate”	Scotland: Threipmuir Reservoir (1996)	EF143264	EF151963
Clade 2A	RGBE SEL834L	*S. pupula*	*S. pupula* agg. “small lanceolate”	United Kingdom: Scotland, Perthshire, Loch Leven 56.18 N 3.34 W 9 (2008)	HQ317116 HQ337594	–
	BLA12	*S. lanceolata*	*S. lanceolata*	Scotland: Blackford Pond (2003)	EF143315	–
	BLA13	*S. lanceolata*	*S. lanceolata*	Scotland: Blackford Pond (2003)	EF143305	EF151978
	RGBE SEL212D	*S. pupula*	*S. pupula* sensu sticto	Scotland: Edinburgh, Dunsapie Loch (2003)	HQ317117 HQ337596	–
	RGBE 32/83	*S. pupula*	*S. pupula* agg. “thick”	Czech Republic: Hradcansky pond. 50.62 N 14.71 E (2007)	HQ317128 HQ337608	–
Clade 2B	DUN8	*S. pupula*	*S. pupula* agg. “small capitate”	Scotland: Dunsapie Loch (2003)	EF143270	–
	DUN7	*S. pupula*	*S. pupula* agg. “small capitate”	Scotland: Dunsapie Loch (2003)	EF143269	EF151961

	RGBE 28/36	*S. pupula*	*S. pupula* agg. cf "marvanii"	Czech Republic: Cerny pond 50.61 N 14.76 E (2007)	HQ317124 HQ337604	–
	RGBE 28/6	*S. pupula*	*S. pupula* agg. cf "marvanii"	Czech Republic: Cerny pond 50.61 N 14.76 E (2007)	HQ317125 HQ337605	–
	RGBE 060	*S. pupula*	*S. pupula* agg. "pseudolanceolate"	Czech Republic: Obectov 49.73 N 16.93 E E (2007)	HQ317127 HQ337607	–
	RGBE 068	*S. pupula*	*S. pupula* agg. "rod"	Czech Republic: Obectov 49.73 N 16.93 E (2007)	HQ317126 HQ337606	–
	RBG2	*S. pupula*	*S. pupula* agg. "little"	Scotland: Royal Botanic Garden Pond (1996)	EF143275	EF151964
	BLA16	*S. pupula*	*S. pupula* agg. "spindle"	Scotland: Blackford Pond (2005)	EF143298	EF151974
	Ak1876	*S.* sp.	*S.* sp.	Japan:Hokkaido, Lake Toro (2020)	LC648444	–
	TCC524	*S. minima*	*S. minima*	France: Reunion island Saint Denis river upstream site AEP socket (2009)	KF959642	–
		S. cf. *minima*	*S.* cf. *minima*	Scotland: Blackford Pond (1997)	EF143279	EF151966
Clade 3	BAL1	*S. blackfordensis*	*S. blackfordensis*	Scotland: Balgavies Loch 56.65 N 2.77 W (1997)	EF143310	–
	(Bfp5x8)F 1-3	*S. blackfordensis*	*S. blackfordensis*	Culture collection BCCM/DCG	JN418669	–
	RBGE BAL7(3)	*S. blackfordensis*	*S. blackfordensis*	United Kingdom: Scotland, Balgavies Loch (1997)	HQ317112 HQ337589	–
	DUN5	*S. blackfordensis*	*S. blackfordensis*	Scotland: Dunsapie Loch (2003)	EF143319	–
	BLA8	*S. blackfordensis*	*S. blackfordensis*	Scotland: Blackford Pond (1997)	EF143290	–
	BLA6	*S. blackfordensis*	*S. blackfordensis*	Scotland: Blackford Pond (1996)	EF143282	EF151969
	BLA7	*S. blackfordensis*	*S. blackfordensis*	Scotland: Blackford Pond (?)	EF143283	–
	AUS1	*S. pupula*	*S. pupula* agg. "southern capitate"	Australia: Hacking River 34°04′22″ S, 151°03′27″ E (2001)	EF143312	EF151982
Clade 3	AUS2	*S. pupula*	*S. pupula* agg. "southern capitate"	Australia: Hacking River (2001)	EF143306	–
	AUS3	*S. pupula*	*S. pupula* agg. "southern capitate"	Australia: Hacking River (2001)	EF143278	–

	SEL793Tm	*S. pupula*	*S. pupula* agg. “gross”	United Kingdom: Scotland, Threipmuir Reservoir (2008)	HQ317131 HQ337613	–
	THR7	*S. pupula*	*S. pupula* agg. cf. “capitata”	Scotland: Threipmuir Reservoir (1998)	EF143302	EF151976
	E4391	*S.* sp.	*S. caput* K.M. Evans & D.G. Mann	United Kingdom: Scotland, Loch Tulla 56.55 N 4.75 W (2003)	HQ317114 HQ337591	–
	AUS4	*S. pupula*	*S. pupula* agg. “southern pseudocapitate”	Australia: Hacking River (2001)	EF143317	EF151983
	RGBE BB92	*S. pupula*	*S. pupula* agg. “australis”	Australia: Victoria, Melbourne, Bolin Billabong 37.77 S 145.08 E (2007)	HQ317118 HQ337597	–
	THR12	*S. pupula*	*S. pupula* agg. “pseudocapitate”	Scotland: Threipmuir Reservoir (1998)	EF143292	–
	RGBE (L845 + Thr42)F1	*S. pupula*	*S. pupula* agg. “pseudocapitate”	United Kingdom: Scotland (1999)	HQ317115 HQ337593	–
	F1	*S. pupula*	*S. pupula* agg. “pseudocapitate”	F_1 clone (1999)	EF143291	–
	US1	*S. pupula*	*S. pupula* agg. “pseudocapitate”	USA: Black Dog Lake Creek, Dakota County, Minnesota (1996)	EF143314	–
Clade 3	KEL–2015	*S.* sp.	*S.* sp.	USA: Georgia, Milledgeville, south of airport 33.1472 N 83.2505 W (2013)	KM999065	–
	BLA9	*S. pupula*	*S. capitata*	Scotland: Blackford Pond (1996)	AY571746	–
	AFR1	*S. pupula*	*S. pupula* agg. “afro”	Tanzania: Ngoro Ngoro crater (2002)	EF143300	EF151975
	BEL2	*S. pupula*	*S. pupula* agg. cf. “obese”	Belgium: Kranepoel (51°04′ N, 3°29′ E) (2001)	EF143266	EF151973
	D06_060	*S. pupula*	*S. pupula* sensu lato	Germany, small river (52.52 N 13.47 E) (2004)	KM084945	–
	D06_110	*S. pupula*	*S. pupula* sensu lato	Germany, small river 52.52 N 13.47 E” (2004)	KM084958	–
	RGBE Pd131	*S. pupula*	*S. pupula* agg. “capitata–like”	Australia: Victoria, Lake Purrumbete 38.28 S 143.21 E (2007)	HQ317122 HQ337601	–

	UTEX LB FD112	*S. pupula*	*S. pupula* agg. “new US”	USA: Iowa (1991)	HQ317123 HQ337602	–
Clade 4	THR6	*S. laevissima*	*S. laevissima*	Scotland: Threipmuir Reservoir (2003)	EF143293	–
	THR5	*S. laevissima*	*S. laevissima*	Scotland: Threipmuir Reservoir (2003)	EF143307	–
	THR4	*S. laevissima*	*S. laevissima*	Scotland: Threipmuir Reservoir (2003)	EF143309	EF151981
	THR3	*S. laevissima*	*S. laevissima*	Scotland: Threipmuir Reservoir (2003)	EF143313	–
	THR2	*S. laevissima*	*S. laevissima*	Scotland: Threipmuir Reservoir (1996)	EF143265	–
	THR1	*S. laevissima*	*S. laevissima*	Scotland: Threipmuir Reservoir (1996)	EF143263	EF151979
	KEL-2015 clone JAR89_B2 Run14	*S.* cf. *laevissima*	*S.* cf. *laevissima*	Canada: Ontario, Petawawa Forestry Station 45.992966 N 77.399194 W (2014)	KM999088	–
	B385	*S. balashovae*	S. *balashovae*	Russia: Lake Frolikha, Baikal region (2012)	MG515209	MG515210
	HYU-D014	*S. seminulum*	*S. seminulum*	South Korea, river stone	MK576038	–
	D06_006	*S. seminulum*	*S. seminulum*	Germany, small river 52.52 N 13.47 E (2004)	KM084937	–
	Styx 1 LCR-S-19-1-1	*S.* sp.	*S. styxii*	New Zealand, Styx River	JQ610174	–
	TCC461	*S. seminulum*	*S. seminulum*	France: Mayotte, river (2009)	KC736613	–
	TM 37	*S.* cf. *seminulum*	*S.* cf. *seminulum*	Scotland: Threipmuir Reservoir (1997)	EF143280	EF151967
